# Effect of glycemic index and carbohydrate intake on kidney function in healthy adults

**DOI:** 10.1186/s12882-016-0288-5

**Published:** 2016-07-08

**Authors:** Stephen P. Juraschek, Alex R. Chang, Lawrence J. Appel, Cheryl A. M. Anderson, Deidra C. Crews, Letitia Thomas, Jeanne Charleston, Edgar R. Miller

**Affiliations:** The Johns Hopkins School of Medicine, The Johns Hopkins Bloomberg School of Public Health, and The Welch Center for Prevention, Epidemiology and Clinical Research, Johns Hopkins Medical Institutions, 2024 East Monument Street, Suite 1–500, Baltimore, MD 21205 USA; Geisinger Health System, Danville, PA USA; University of California San Diego, San Diego, CA USA

**Keywords:** β2-microglobulin, Carbohydrate, Clinical trial, Creatinine, Cystatin C, Diet, Estimated glomerular filtration rate, Glycemic index

## Abstract

**Background:**

Replacing carbohydrate with protein acutely increases glomerular filtration rate (GFR) but is associated with faster, long-term kidney disease progression. The effects of carbohydrate type (i.e. glycemic index, GI) on kidney function are unknown.

**Methods:**

We conducted an ancillary study of a randomized, crossover feeding trial in overweight/obese adults without diabetes or kidney disease (*N* = 163). Participants were fed each of four healthy, DASH-like diets for 5 weeks, separated by 2-week washout periods. Weight was kept constant. The four diets were: high GI (GI ≥65) with high %carb (58 % kcal) (reference diet), low GI (≤45) with low %carb (40 % kcal), low GI with high %carb; and high GI with low %carb. Plasma was collected at baseline and after each feeding period. Study outcomes were cystatin C, β2-microglobulin (β2M), and estimated GFR based on cystatin C (eGFRcys).

**Results:**

Mean (SD) age was 52 (11) years; 52 % were women; 50 % were black. At baseline, mean (SD) cystatin C, β2M, and eGFRcys were 0.8 (0.1) mg/L, 1.9 (0.4) mg/L, and 104 (16) mL/min/1.73 m^2^. Compared to the high GI/high %carb diet, reducing GI, %carb, or both increased eGFRcys by 1.9 mL/min/1.73 m^2^ (95 % CI: 1.1, 2.7; *P* < 0.001), 3.0 mL/min/1.73 m^2^ (1.9, 4.0; *P* < 0.001), and 4.5 mL/min/1.73 m^2^ (3.5, 5.4; *P* < 0.001), respectively. Increases in eGFRcys from reducing GI were significantly associated with increases in eGFRcys from reducing %carb (*P* < 0.001). Results for cystatin C and β2M reflected eGFRcys.

**Conclusions:**

Reducing GI increased GFR. Reducing %carb by increasing calories from protein and fat, also increased GFR. Future studies on GI should examine the long-term effects of this increase in GFR on kidney injury markers and clinical outcomes.

**Trial registration:**

Clinical Trials.gov, number: NCT00608049 (first registered January 23, 2008)

**Electronic supplementary material:**

The online version of this article (doi:10.1186/s12882-016-0288-5) contains supplementary material, which is available to authorized users.

## Background

Replacing dietary carbohydrates with protein increases glomerular filtration rate (GFR) acutely and is associated with faster chronic kidney disease (CKD) progression over the long-term [1–3]. Whether carbohydrate quality affects GFR is unknown. Glycemic index (GI) is a measure of carbohydrate quality, i.e. the amount of glucose released into circulation from carbohydrate-based foods [4]. GI is determined by comparing the area under a 2-h glucose curve measured after consumption of a standardized amount of carbohydrates from two food items (the food of interest and a reference food) [5]. Previous observational studies demonstrated that higher GI is associated with prevalent CKD [6]. However, this association has not been examined in a trial setting and could have implications for CKD prevention.

In this analysis of data from “The Effect of Amount and Type of Dietary Carbohydrates on Risk for Cardiovascular Heart Disease and Diabetes Study” (OMNICARB), we determined the effects of reducing GI or the proportion of carbohydrates (%carb) on GFR in relatively healthy adults, using a cystatin C estimating equation. We further estimated GFR with a creatinine-based equation for comparison. We hypothesized that reducing GI would increase GFR as a result of metabolic changes, and that reducing %carb (i.e. increasing the proportion of protein) would increase GFR. We reported this relationship to change in protein intake in an earlier publication from a different feeding study (OMNI-HEART) [7]. A cystatin C-based equation, rather than a creatinine-based equation, was our primary outcome because of a concern that changes in dietary protein intake would influence serum creatinine levels and interfere with accurate estimation of kidney function [7].

## Methods

The rationale, design, and main effects of OMNICARB were described previously [8]. The current ancillary study utilized stored plasma specimens to examine the effects of amount and type of carbohydrates on markers of kidney function.

OMNICARB was a randomized, crossover trial, comprising 4-dietary interventions: high GI (GI ≥65) with high %carb (58 % kcal from carbohydrates) (reference diet), low GI (GI ≤45) with low %carb (40 % kcal from carbohydrates), low GI with high %carb; and high GI with low %carb [8]. Other key nutrients were similar across the four diets (Table 1). By design, the diets were healthy using the core nutrient profile of the DASH diet [9]. Each diet was isocaloric, was prepared with commonly available foods, and was low in saturated fat, cholesterol, and sodium, but rich in fruits, vegetables, fiber, potassium, and other minerals. Note that the low %carb diets were also higher in % protein.

**Table 1:** Nutrient composition of the four diets used in OmniCarb^a^

Dietary pattern	High carbohydrate, high glycemic index	High carbohydrate, low glycemic index	Low carbohydrate, high glycemic index	Low carbohydrate, low glycemic index
Energy, kcal	2011	1998	2011	1993
Glycemic Index	66	41	65	40
Carbohydrates, % kcal	58	57	41	40
Protein, % kcal	16	16	23	23
Fat, % kcal	27	27	37	37
Saturated, % kcal	6	6	7	7
Monounsaturated, % kcal	12	13	18	19
Polyunsaturated, % kcal	7	8	10	10
Fiber, g	32	37	29	33
Cholesterol, mg	90	89	170	163
Calcium, mg	1032	1051	993	995
Potassium, mg	3963	4103	3949	4026
Sodium, mg	2245	2211	2305	2199
Magnesium, mg	462	429	468	440

^a^Estimated from food analysis software (ESHA Food Processor SQL, version 10.2, ESHA Research)

### Participant recruitment

Trial participants were adult men and women, residing in and around Boston, Massachusetts, and Baltimore, Maryland. Participants were ≥30 years, had a body mass index ≥25 kg/m^2^ with a systolic BP of 120–159 and a diastolic BP <100 mmHg. Persons with a prior diagnosis of diabetes or cardiovascular disease were excluded [8]. Persons were also excluded if they had renal insufficiency on screening labs, based on an estimated GFR <40 ml/min per 1.73 m^2^. Similarly, participants were deemed eligible for inclusion if baseline systolic blood pressure was 120–159 mmHg and diastolic blood pressure was <100 mmHg (mean over three screening visits). Anyone with stage 2 hypertension (systolic blood pressure > 160 or diastolic blood pressure > 100 mmHg) based on the mean of three screening visits were excluded. Anyone with a mean systolic blood pressure >170 or diastolic blood pressure >105 mmHg at any one visit were also excluded [8]. Institutional Review Boards approved the study protocol, and all participants provided written informed consent.

### Controlled feeding

Controlled feeding began in August 2008 and was completed in December 2010. Participants were randomly assigned to 1 of 8 dietary sequences of the 4 diets [8]. Feeding periods lasted for 5 weeks, separated by 2-week washout periods. Calorie targets were determined for each participant based on body size, sex, and physical activity level. Participants were provided all of their foods and caloric intake was adjusted to maintain weight within 2 % of participants’ baseline values. Participants were encouraged to maintain the same activity levels and alcohol consumption throughout the study. Every day, participants ate at least one meal at the study site and meal attendance and meal consumption were monitored. Participants also completed a diary in which they listed any consumption of non-protocol foods. Biochemical markers of dietary compliance were urine urea nitrogen, urine creatinine, urine sodium, and urine potassium.

### Measurement of outcomes

Laboratory specimens were collected for each participant at baseline and at the completion of each 5-week feeding period. Cystatin C, β2 microglobulin (β2M), and creatinine were measured in plasma samples that were collected in tubes, centrifuged, and stored at −70 °C. Cystatin C and β2M were used to estimate kidney function, whereas plasma creatinine and estimated glomerular filtration rate based on creatinine (eGFRcreat) were measured for purposes of comparison. When compared to creatinine, these markers are less influenced by factors know to affect creatinine such as race [10] and diet (e.g. protein intake) [7]. Both cystatin C and β2M were measured with particle-enhanced immunonephelometric assays (N Latex Cystatin C assay and N Latex β2M assay, Dade Behring, IL). Inter-assay coefficients of variation for cystatin C and β2M were 2.0 % (mean 0.917 mg/L) and 1.8 % (mean 2.184 mg/L), respectively. Creatinine was measured in stored plasma specimens using standardized laboratory assays with an inter-assay coefficient of variation of 6.6 % (mean 0.653 mg/dL). Glomerular filtration rate was estimated using the Chronic Kidney Disease Epidemiology Collaboration cystatin C-based equation (eGFRcys) [11] and the Chronic Kidney Disease Epidemiology Collaboration creatinine-based GFR equation (eGFRcreat) [12]. As opposed to other estimating equations, these equations were used because they were developed in a population sample that included individuals, who like the participants of the OMNICARB trial, did not have chronic kidney disease (CKD).

### Other covariate measurements and definitions

Additional laboratory and physical exam covariates were determined as follows. Body mass index (BMI) was calculated using baseline height and weight measurements and was dichotomized as 25–29.9 kg/m^2^ or ≥30 kg/m^2^. Waist circumference (cm) was measured at the level of the umbilicus. Fasting glucose and insulin were measured at baseline and at the end of each feeding period. The homeostasis model assessment index (HOMA) was calculated using HOMA = [(fasting serum insulin concentration in μU/mL) x (fasting serum glucose concentration in mmol/L)]/22.5 and dichotomized based on the baseline median value of ≥1.48. Similarly, traditional assays were used to measure total triglycerides and HDL cholesterol. LDL cholesterol levels were estimated by the Friedewald equation [13]. Triglycerides were dichotomized using the baseline median value of 83.8 mg/dL. Uric acid and high sensitivity C-reactive protein were also measured in plasma collected at baseline and the end of each feeding period.

Baseline hypertension status (yes or no) was determined by an average of 3 baseline blood pressure measurements in which systolic blood pressure was >140 mmHg or diastolic blood pressure was >90 mmHg. No participants were taking antihypertensive medications. Blood pressure was also measured at the end of each feeding period as the average of 3 measurements by trained and certified staff using a validated automated oscillometric OMRON 907 device.

### Mediators of glomerular filtration

At baseline and at the end of each feeding period, we assessed potential mediators of glomerular filtration, including markers of glucose homeostasis (fasting glucose, insulin, triglycerides), endothelial function (uric acid, systolic BP, diastolic BP), and inflammation (high sensitivity C-reactive protein).

### Statistical analysis

The primary outcomes examined in this study were cystatin C, β2M, and eGFRcys. We estimated change from baseline for each of the 4 diets. Using the high %carb, high GI diet as a reference, we then calculated the effects of reducing GI alone, %carb alone, or both GI and %carb for each outcome and generated forest plots for visual representation. In addition, we performed stratified analyses by covariates known to be associated with insulin resistance, namely, race (non-Hispanic black versus white), baseline hypertension status, baseline triglycerides, baseline BMI, and baseline HOMA. All of the above comparisons were performed via generalized estimating equation (GEE) regression models, using a Huber and White robust variance estimator [14], which assumed an exchangeable working correlation matrix. *P*-values for each stratum were generated using interaction terms. The above analyses were repeated for creatinine and eGFRcreat for comparison.

We also used linear regression to determine whether the change in eGFRcys which resulted from reducing GI was associated with change in eGFRcys which resulted from reducing %carb. A positive association would suggest a common mechanism of action for GI and %carb on kidney function. We further performed a mediation analysis of eGFRcys, adjusting for concurrent changes in markers of glucose homeostasis (fasting glucose, insulin, triglycerides), endothelial function (uric acid, systolic blood pressure, diastolic blood pressure), and inflammation (high sensitivity C-reactive protein), all thought to influence GFR.

For dietary compliance measures, namely, urine urea nitrogen, urine creatinine, urine sodium, and urine potassium, we performed baseline and between-diet comparisons only, using similar models described above. A sensitivity analysis using feeding period 1 alone was conducted to eliminate potential carryover effects. We also performed a sensitivity analysis examining every possible dietary comparison grouped by GI, %carb, or simultaneous changes in both GI and %carb. The purpose of this analysis was to confirm our primary comparison between diets, which utilized the high GI, high %carb diet as our reference group. All analyses were performed in STATA version 11.1 (Stata Corporation, College Station, TX, USA). Statistical significance was defined as *P* ≤ 0.05. Missing data specimens were excluded from analyses as they were few (*N* = 4) and evenly distributed between feeding periods and diets.

## Results

Baseline characteristics of the randomized, study population are shown in Table 2. Overall mean age was 52.1 ± 11.4 years; 52 % of the participants were women, and 50 % were non-Hispanic blacks. Furthermore, 56 % of participants were obese, and 26 % had hypertension.

**Table 2:** Baseline characteristics of trial participants (*N* = 163), mean ± SD or No. (%)

Age, y	52.1 ± 11.4
Women	85 (52)
Race	
Non-Hispanic White	66 (40)
Non-Hispanic Black	82 (50)
Hispanic	11 (7)
Asian	4 (2)
Body mass index, kg/m^2^	32.3 ± 5.5
Body mass index	
25–29.9	71 (44)
≥ 30	92 (56)
Waist circumference, cm	104.4 ± 13.5
Fasting glucose, mg/dL	97.3 ± 13.6
Fasting insulin, μU/mL	7.7 ± 5.8
Homeostasis model assessment (HOMA), units	1.9 ± 1.6
Triglycerides, mg/dL	104.6 ± 67.1
HDL cholesterol, mg/dL	58.3 ± 16.0
LDL cholesterol, mg/dL	153.0 ± 42.1
Systolic blood pressure, mm Hg	132.0 ± 9.1
Diastolic blood pressure, mm Hg	80.0 ± 7.5
Baseline hypertension status^a^	
No hypertension	120 (74)
Hypertension	43 (26)
Uric acid, mg/dL^b^	4.7 ± 1.2
High sensivity C-reactive protein, mg/L^b^	3.2 ± 4.0

^a^Defined as baseline SBP ≥ 140 or DBP ≥ 90 mmHg

^b^Only measured in 159 participants due to lost specimens

End-of-period measures were compared to baseline measures (Table 3). Cystatin C was significantly greater during the high GI, high %carb diet (*P* =0.04) and significantly lower during the high GI/low %carb, and low GI/low %carb diets (both *P* ≤0.001). β2M was significantly lower during the low GI/low %carb diet (*P* =0.002). While eGFRcys decreased significantly ~1 mL/min/1.73 m^2^ during the high GI/high %carb diet (*P* =0.02), it increased significantly during the high GI/low %carb (~2 mL/min/1.73 m^2^; *P* =0.002) and low GI/low %carb diets (~3 mL/min/1.73 m^2^; *P* <0.001). Creatinine did not change significantly during any dietary period throughout the trial. There was no significant difference between end-of-period eGFRcreat and baseline during any of the four diets.

**Table 3:** Plasma markers of kidney function and urine markers of compliance, *N* = 159

		Mean (95 % Confidence Interval) change from baseline by diet							
		High carbohydrate, high glycemic index diet		High carbohydrate, low glycemic index diet		Low carbohydrate, high glycemic index diet		Low carbohydrate, low glycemic index diet	
	Baseline Mean (SD)	Difference, 95 % CI	*P*	Difference, 95 % CI	*P*	Difference, 95 % CI	*P*	Difference, 95 % CI	*P*
Cystatin C, mg/L	0.8 (0.1)	0.01 (0.00, 0.02)	0.04	−0.01 (−0.02, 0.00)	0.13	−0.02 (−0.03,–0.01)	0.001	−0.03 (−0.04,–0.03)	<0.001
B2-microglobulin, mg/L	1.9 (0.4)	0.04 (−0.01, 0.08)	0.10	0.00 (−0.04, 0.03)	0.86	−0.04 (−0.07, 0.00)	0.06	−0.05 (−0.09,–0.02)	0.002
Creatinine, mg/dL	0.8 (0.2)	−0.01 (−0.02, 0.00)	0.15	−0.01 (−0.03, 0.00)	0.14	0.01 (−0.01, 0.03)	0.21	−0.01 (−0.02, 0.01)	0.25
eGFRcys, mL/min/1.73 m^2^	104.2 (16.0)	−1.19 (−2.19,–0.18)	0.02	0.71 (−0.20, 1.63)	0.13	1.78 (0.67, 2.90)	0.002	3.28 (2.32, 4.24)	<0.001
eGFRcreat, mL/min/1.73 m^2^	100.1 (16.4)	0.60 (−0.71, 1.91)	0.37	0.83 (−0.57, 2.23)	0.24	−0.87 (−2.44, 0.69)	0.27	0.67 (−0.82, 2.15)	0.38
Urine nitrogen, mg/d	10116.4 (4767.7)	−13.0 (−769.4, 743.5)	0.97	257.8 (−500.7, 1016.2)	0.51	3734.9 (2821.3, 4648.4)	<0.001	3852.6 (2946.7, 4758.5)	<0.001
Urine creatinine, mg/d	1463.9 (592.7)	−71.2 (−160.0, 17.7)	0.12	−117.3 (−204.6, −30.0)	0.01	37.5 (−59.4, 134.4)	0.45	−9.3 (−109.0, 90.3)	0.86
Urine sodium, mmol/d	152.9 (77.7)	−31.4 (−44.1, −18.7)	<0.001	−38.6 (−51.3, −25.9)	<0.001	−29.5 (−41.6, −17.5)	<0.001	−35.5 (−48.9, −22.1)	<0.001
Urine potassium, mmol/d	56.9 (26.2)	19.8 (14.6, 24.9)	<0.001	19.7 (14.6, 24.8)	<0.001	19.3 (14.3, 24.2)	<0.001	16.9 (11.0, 22.7)	<0.001

Note: eGFRcys represents cystatin C-based estimated glomerular filtration rate; eGFRcreat represents creatinine-based estimated glomerular filtration rate

When compared with baseline, urine urea nitrogen was significantly higher during the low %carb diets (*P* <0.001 for high GI/low %carb and *P* <0.001 for low GI/low %carb) consistent with the higher protein composition of these diets. Urine creatinine tended to decrease during the high %carb diets (*P* =0.12 for high GI/high %carb and *P* =0.01 for low GI/high %carb), consistent with the diets’ lower protein composition (16 % vs. 23 %). For all diets, urine sodium significantly decreased and urine potassium increased (all *P*-values <0.001). This was expected given the low sodium and high fruits and vegetable-based foods inherent in all the OMNICARB diets.

**Fig. 1:**
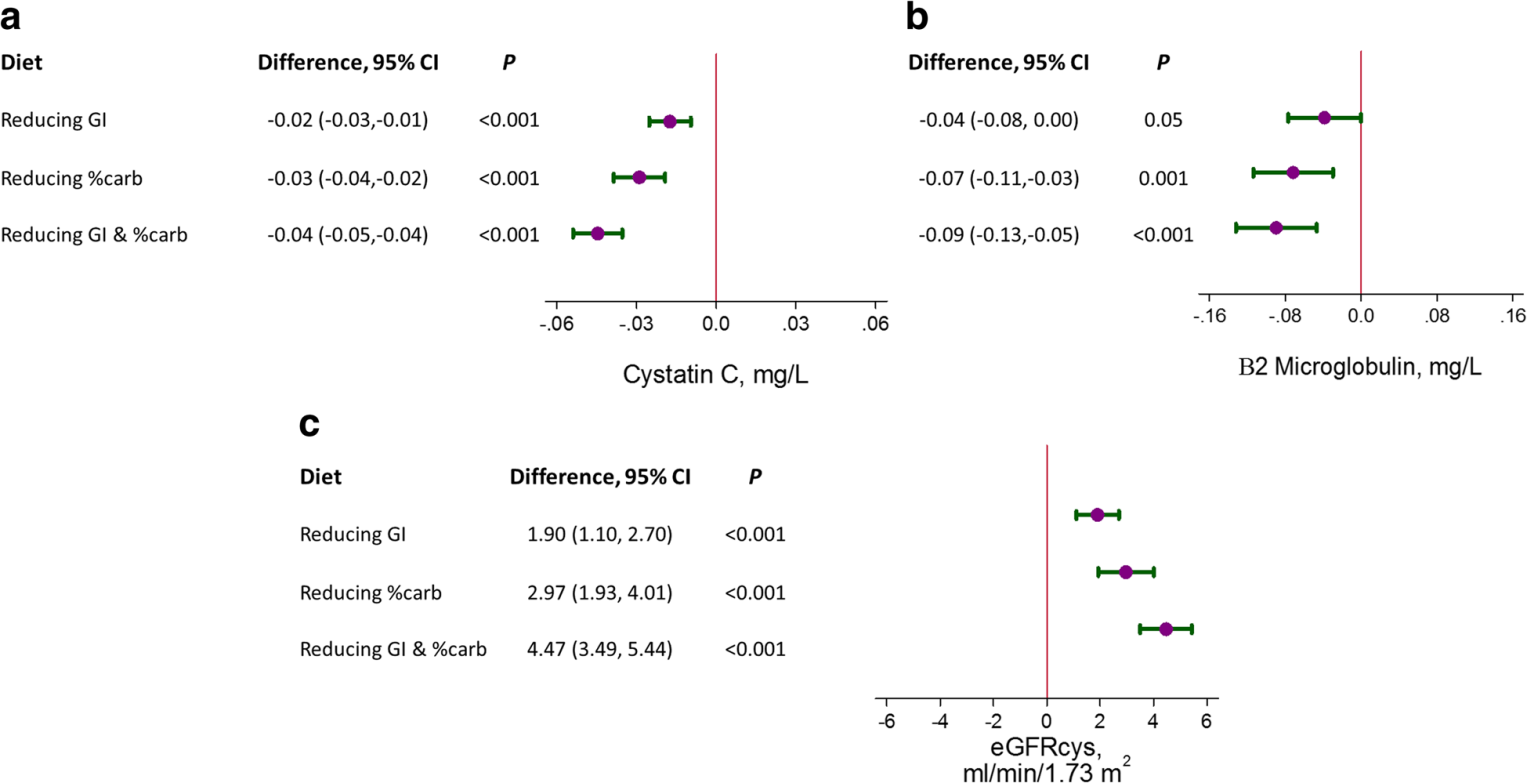
The effects (95 % confidence intervals) of reducing glycemic index (GI), reducing the proportion of carbohydrates (%carb), or reducing both GI and %carb on: **a** cystatin C (mg/L), **b** β2-microglobulin (mg/L), and **c** cystatin C-based estimated glomerular filtration rate (eGFRcys) (mL/min/1.73 m^2^) measured at the end of each feeding period. The reference diet was the high GI/high %carb diet

The effects of reducing GI or %carb on β2M, cystatin C, and eGFRcys were compared across diets, using the high GI,/high %carb diet as a reference (Fig. 1a-c). Reducing GI, %carb, or both GI and %carb significantly reduced cystatin C (*P* <0.001 for all three) and β2M (*P* <0.001 for all three). Each of the diets significantly increased eGFRcys (*P* <0.001 for all three). Furthermore, the magnitude of effect was higher, ranging from ~2 mL/min/1.73 m^2^ (reduced GI) and ~3 mL/min/1.73 m^2^ (reduced %carb) to 4.5 mL/min/1.73 m^2^, when both GI and %carb were reduced.

The effects of dietary carbohydrates on plasma creatinine and eGFRcreat were also assessed (Additional file 1: Figure S1A-B). While reducing %carb increased serum creatinine (*P* = 0.001), increased urine creatinine excretion (*P* = 0.007), and decreased eGFRcreat by 1.5 mL/min/1.73 m^2^ (*P* = 0.02), reducing GI or both factors had no effect on serum creatinine, urine creatinine excretion, or eGFRcreat.

In general, the observed effects on kidney markers and GFR described above were confirmed when every possible dietary comparison was performed with the exception of creatinine and eGFRcreat, which demonstrated greater variability from changes in dietary factors (i.e. GI or %carb) (Additional file 1: Table S3).

We compared the diets’ effects on cystatin C, β2M, creatinine, eGFRcys, and eGFRcreat by strata of factors associated with CKD (Additional file 1: Table S1). There was no evidence of interactions by race, hypertension status, baseline triglycerides, BMI, and HOMA on any of the observed dietary effects. A sensitivity analysis utilizing feeding period 1 alone may be found in Additional file 1: Table S2. While the patterns of association were unaltered, our results were attenuated when we restricted our analysis to feeding period 1 only.

Finally, we specifically examined the mechanism by which GI and %carb affected eGFRcys. We found that the renal response to reducing GI was significantly associated with the renal response to reducing %carb (*P* < 0.001) (Additional file 1: Figure S2). Further, the effects of reducing GI or %carb on eGFRcys were only mildly attenuated with adjustment for potential mediators of kidney function, specifically, fasting glucose, insulin, triglycerides, uric acid, systolic blood pressure, and diastolic blood pressure (Table 4).

**Table 4:** Change in estimated glomerular filtration rate based on cystatin C adjusted for fasting glucose, fasting insulin, fasting triglycerides, uric acid, systolic blood pressure, diastolic blood pressure, and high sensitivity C-reactive protein

Reducing glycemic index	Change in eGFRcys (95 % CI)	*P*
eGFRcys, mL/min/1.73 m^2^, unadjusted	1.90 (1.10, 2.70)	<0.001
Adjusted for fasting glucose, insulin, triglycerides	1.82 (1.01, 2.63)	<0.001
Adjusted for uric acid, systolic blood pressure, diastolic blood pressure	1.47 (0.70, 2.23)	<0.001
Adjusted for high sensitivity C-reactive protein	1.89 (1.09, 2.69)	<0.001
Adjusted for all the above	1.38 (0.62, 2.15)	<0.001
Reducing carbohydrate proportion		
eGFRcys, mL/min/1.73 m^2^, unadjusted	2.97 (1.93, 4.01)	<0.001
Adjusted for fasting glucose, insulin, triglycerides	2.51 (1.45, 3.56)	<0.001
Adjusted for uric acid, systolic blood pressure, diastolic blood pressure	3.27 (2.28, 4.25)	<0.001
Adjusted for high sensitivity C-reactive protein	2.96 (1.92, 4.00)	<0.001
Adjusted for all the above	2.84 (1.83, 3.86)	<0.001
Reducing both glycemic index and carbohydrate proportion		
eGFRcys, mL/min/1.73 m^2^, unadjusted	4.47 (3.49, 5.44)	<0.001
Adjusted for fasting glucose, insulin, triglycerides	3.88 (2.84, 4.92)	<0.001
Adjusted for uric acid, systolic blood pressure, diastolic blood pressure	4.14 (3.22, 5.05)	<0.001
Adjusted for high sensitivity C-reactive protein	4.50 (3.54, 5.46)	<0.001
Adjusted for all the above	3.67 (2.70, 4.64)	<0.001

Note: eGFRcys represents estimated glomerular filtration rate-based on cystatin C

## Discussion

For the first time in a trial setting, we demonstrated that reducing GI, i.e. changing the type of carbohydrate, reduced markers of kidney function and increased glomerular filtration. Furthermore, this study confirmed prior observations that reducing %carb (i.e. increasing %protein/fat), increases kidney function. Moreover, an increase in eGFRcys from reducing GI tended to have a similar effect as that observed from reducing %carb, and these effects were additive, such that reducing both GI and %carb increased eGFRcys by 4.5 mL/min/1.73 m^2^. The effects persisted after adjustment for potential mediators of kidney function.

In our study, replacing carbohydrates with fat and protein increased GFR as estimated by cystatin C and β2M. These results are consistent with previous studies that test dietary effects on GFR [7]. Animal and human studies have indicated that dietary protein increases renal blood flow and glomerular filtration rates via increased intraglomerular pressures, leading to progressive glomerular sclerosis, particularly in the setting of decreased nephron mass [15–19]. These effects may be mediated by dietary effects on signaling molecules of the tubuloglomerular feedback system, responsible for arteriolar constriction [20–22]. It is also believed that dietary protein may increase neuronal nitric oxide synthase in the kidney cortex, which relaxes the afferent renal arteriole [23, 24]. Although fat was also higher in the low carbohydrate/high protein diet, there is much less evidence for a relationship between a higher fat diet and hyperfiltration [25], though some have hypothesized that such an association could be mediated through inflammation [26, 27].

This study is among the first to examine the effects of GI on GFR. GI, a metric of carbohydrate quality determined by measuring the amount of glucose released into circulation after consumption of a standard amount of carbohydrates [4], could influence kidney function. Reducing GI has been reported to decrease inflammation [28]. Furthermore, animal models have shown that a high GI diet contributes to glomerular degeneration [29]. Recently, an observational study of 2,600 adults reported a cross-sectional association between dietary GI and an estimated GFR <60 mL/min per 1.73 m^2^ (OR = 1.55 [1.07–2.26]; *P*-trend =0.01), but dietary GI was not associated with incident CKD over a 5-year follow-up period [6]. Contrary to the above studies and our *a priori* hypothesis, we found that a lower GI diet decreased kidney filtration markers and increased eGFRcys. While the exact mechanism for this observation is unknown, several animal models have reported an association between complex carbohydrates (i.e. low GI foods) and improved renal endothelial nitric oxide synthase [30, 31] or decreased leukocyte binding with glomerular endothelial cells [32]. Whether or not the increased GFR observed in conjunction with the low GI diets contributes adversely to clinical outcomes long-term remains to be determined.

Our study suggests that reducing GI or %carb share a common pathway for affecting eGFRcys (Additional file 1: Figure S2). These changes are independent of markers of plasma glucose homeostasis, endothelial function, and inflammation, implying that dietary carbohydrates may have a direct effect on glomerular filtration [33]. We suspect that these short-term increases in glomerular filtration from reducing GI and/or %carb, represent hyperfiltration, a maladaptive response to abnormal renal hemodynamics and a harbinger of kidney injury and kidney disease progression [34, 35]. The long-term impact of these short-term changes in eGFRcys on clinical outcomes is unknown. Novel markers of kidney injury in urine [36] may help elucidate the benefit or harm of dietary carbohydrates in future studies.

Cystatin C and β2-microglobulin (β2M) are low molecular mass plasma proteins that are alternative markers to creatinine for estimating GFR and predicting CKD progression [37–39]. Both cystatin C and β2M are generated at a constant rate by all nucleated cells, freely filtered by glomeruli, then reabsorbed and metabolized by the proximal tubule with minimal reentry to the bloodstream [40–43]. Serum creatinine reflects GFR, but as a byproduct of protein metabolism it is influenced by dietary protein [7] while cystatin C and β2M do not seem to be influenced by dietary protein [7, 16, 39]. Hence, these markers represent an attractive alternative to creatinine for quantifying GFR in feeding studies that involve changes in dietary protein [7].

In this trial, both cystatin C and β2M were affected by OMNICARB diets in a similar fashion, that is, lower %carb/higher protein or lower GI decreased plasma concentrations of both cystatin C and β2M. In contrast, creatinine did not decline with lower GI and further increased during the low %carb (higher protein)/high GI diet. In absence of measured GFR, this could be interpreted as either non-GFR effects on both cystatin C and β2M or non-GFR effects on creatinine. These observed effects had important ramifications on estimated GFR, which was significantly increased when estimated using cystatin C, but null or decreased when estimated using creatinine. Given the independent biosynthetic pathways of each of these three markers, it is more probable that non-GFR effects would influence one rather than two markers [10], that is in this case, creatinine versus both cystatin C and β2M. This underscores the value of non-traditional markers in better estimating changes in GFR [10, 44].

This study has limitations. The feeding periods were too brief to observe dietary effects on clinical events. Furthermore, the study did not include patients with diabetes or CKD, populations that might demonstrate greater changes in kidney function from alterations in dietary carbohydrates. Moreover, the diets were all DASH-like, healthful diets. It is possible that greater effects on kidney function would be observed if the reference diet was a typical American diet. Finally, it should be noted that by design reductions in %carb intake were coupled with increases in both fat and protein to keep overall caloric intake constant between diets. Thus, it would be inaccurate to conclude that reducing %carb alone increased kidney function. A better interpretation would be that replacing calories from carbohydrates with calories from protein and fat increased kidney function.

This study also has strengths, including being an ancillary of a randomized trial with a diverse population and high follow-up rates that obtained repeat measures of kidney function. Diets were tightly controlled and isocaloric, eliminating the potential effect of weight change on outcomes. Furthermore, we utilized alternative kidney markers instead of creatinine, which permitted more accurate assessments of diet-induced changes in GFR [7].

## Conclusion

In conclusion, we found that both a low GI and low %carb (i.e. higher protein/fat) diets increased GFR, which possibly reflects hyperfiltration or risk of kidney injury. At this point it is unclear whether the increase in GFR caused by low GI diets would be associated with adverse clinical outcomes. This should be an important focus of future studies on GI and kidney function.

## Additional file

Additional file 1:
**Figure S1.** The effects (95% confidence intervals) of reducing glycemic index (GI), reducing the proportion of carbohydrates (%carb), or reducing both GI and %carb on: (A) creatinine (mg/dL) and (B) creatinine-based estimated glomerular filtration rate (eGFRcreat) (mL/min/1.73 m2) measured at the end of each feeding period. **Figure S2.** Scatter plot of the change in cystatin C-based estimated glomerular filtration rate (eGFRcys) (mL/min/1.73 m2) from reducing glycemic index (y-axis) versus reducing proportion of carbohydrate (x-axis). A linear regression line (dashed line) overlays the data (P-value of the coefficient was < 0.001). **Table S1.** Baseline change in glomerular filtration markers by subgroup (reference diet is the high glycemic index, high carbohydrate diet). **Table S2.** Between Diet Comparison Restricted to Visit 1 Only. **Table S3.** All Between Diet Comparisons. (DOCX 165 kb)

## Abbreviations

OMNICARB: The Effect of Amount and Type of Dietary Carbohydrates on Risk for Cardiovascular Heart Disease and Diabetes Study
CG: The high carbohydrate, high glycemic index diet
cG: The low carbohydrate, high glycemic index diet
Cg: The high carbohydrate, low glycemic index diet
cg: The low carbohydrate, low glycemic index diet
GFR: Glomerular filtration rate
β2M: β2-microglobulin
BMI: Body mass index
LDL: Low density lipoprotein
HDL: High density lipoprotein
SBP: Systolic blood pressure
DBP: Diastolic blood pressure
DASH: Dietary approaches to stop hypertension
GEE: Generalized estimating equation
CI: Confidence interval
