# Supplementary material for: Effect of glycemic index and carbohydrate intake on kidney function in healthy adults
Source: BMC Nephrol. 2016 Jul 8;17:70. doi: 10.1186/s12882-016-0288-5 (PMC4938908; doi:10.1186/s12882-016-0288-5)
Supplement: Additional file 1: — Figure S1. The effects (95% confidence intervals) of reducing glycemic index (GI), reducing the proportion of carbohydrates (%carb), or reducing both GI and %carb on: (A) creatinine (mg/dL) and (B) creatinine-based estimated glomerular filtration rate (eGFRcreat) (mL/min/1.73 m2) measured at the end of each feeding period. Figure S2. Scatter plot of the change in cystatin C-based estimated glomerular filtration rate (eGFRcys) (mL/min/1.73 m2) from reducing glycemic index (y-axis) versus reducing proportion of carbohydrate (x-axis). A linear regression line (dashed line) overlays the data (P-value of the coefficient was < 0.001). Table S1. Baseline change in glomerular filtration markers by subgroup (reference diet is the high glycemic index, high carbohydrate diet). Table S2. Between Diet Comparison Restricted to Visit 1 Only. Table S3. All Between Diet Comparisons. (DOCX 165 kb) [file 12882_2016_288_MOESM1_ESM.docx]

**Supplemental Material**

Supplement Figures S1-S2

and

Supplement Tables S1-S3

**
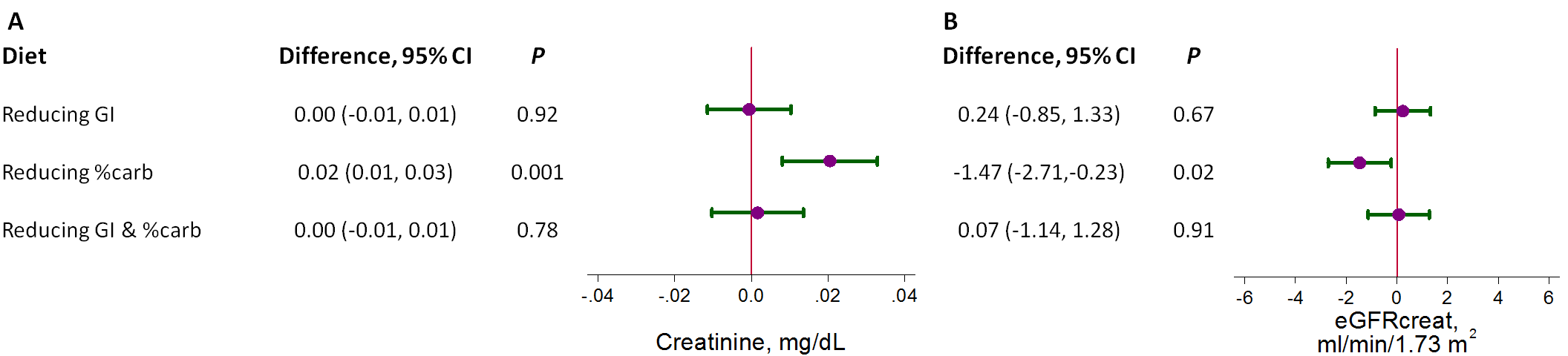
**

**Supplement Figure S1.** The effects (95% confidence intervals) of reducing glycemic index (GI), reducing the proportion of carbohydrates (%carb), or reducing both GI and %carb on: (**A**) creatinine (mg/dL) and (**B**) creatinine-based estimated glomerular filtration rate (eGFRcreat) (mL/min/1.73 m^2^) measured at the end of each feeding period.

**Supplement Figure S2.** Scatter plot of the change in cystatin C-based estimated glomerular filtration rate (eGFRcys) (mL/min/1.73 m^2^) from reducing glycemic index (y-axis) versus reducing proportion of carbohydrate (x-axis). A linear regression line (dashed line) overlays the data (*P*-value of the coefficient was < 0.001).

**Supplemental Tables**

| **Supplement Table S1. Baseline change in glomerular filtration markers by subgroup (reference diet is the high glycemic index, high carbohydrate diet)** | | | | | | | | | | | | | | |
| --- | --- | --- | --- | --- | --- | --- | --- | --- | --- | --- | --- | --- | --- | --- |
|  |  |  |  | **Mean (95% Confidence Interval) Change from Baseline by Diet** | | | | | | | | | | |
|  |  |  |  | **Reducing Glycemic Index** | | |  | **Reducing Carbohydrate** | | |  | **Reducing both Glycemic Index and Carbohydrate** | | |
|  |  |  | **Baseline No.** | **Difference, 95% CI** |  | ***P*** |  | **Difference, 95% CI** |  | ***P*** |  | **Difference, 95% CI** |  | ***P*** |
| **Cystatin C, mg/L** | | |  |  |  |  |  |  |  |  |  |  |  |  |
|  | Non-Hispanic | |  |  |  |  |  |  |  |  |  |  |  |  |
|  |  | White | 65 | -0.02 (-0.03,-0.01) |  | 0.79 |  | -0.03 (-0.05,-0.02) |  | 0.39 |  | -0.04 (-0.06,-0.03) |  | 0.79 |
|  |  | Black | 82 | -0.02 (-0.03,-0.01) |  |  |  | -0.03 (-0.04,-0.01) |  |  |  | -0.05 (-0.06,-0.03) |  |  |
|  | Hypertension status | |  |  |  |  |  |  |  |  |  |  |  |  |
|  |  | No | 117 | -0.02 (-0.03,-0.01) |  | 0.81 |  | -0.03 (-0.04,-0.02) |  | 0.95 |  | -0.04 (-0.05,-0.03) |  | 0.41 |
|  |  | Yes | 42 | -0.02 (-0.03, 0.00) |  |  |  | -0.03 (-0.05,-0.01) |  |  |  | -0.05 (-0.07,-0.03) |  |  |
|  | Baseline Triglycerides, mg/dl | |  |  |  |  |  |  |  |  |  |  |  |  |
|  |  | ≤ 83.8 | 80 | -0.02 (-0.03,-0.01) |  | 0.14 |  | -0.02 (-0.04,-0.01) |  | 0.38 |  | -0.05 (-0.06,-0.03) |  | 0.66 |
|  |  | > 83.8 | 79 | -0.01 (-0.02,-0.00) |  |  |  | -0.03 (-0.05,-0.02) |  |  |  | -0.04 (-0.06,-0.03) |  |  |
|  | Body mass index, kg/m^2^ | |  |  |  |  |  |  |  |  |  |  |  |  |
|  |  | 25-29.9 | 69 | -0.02 (-0.03,-0.01) |  | 0.83 |  | -0.03 (-0.05,-0.02) |  | 0.36 |  | -0.05 (-0.07,-0.04) |  | 0.14 |
|  |  | 30+ | 90 | -0.02 (-0.03,-0.01) |  |  |  | -0.03 (-0.04,-0.01) |  |  |  | -0.04 (-0.05,-0.03) |  |  |
|  | Homeostasis Model Assessment (HOMA) | |  |  |  |  |  |  |  |  |  |  |  |  |
|  |  | ≤1.48 | 80 | -0.01 (-0.02,-0.00) |  | 0.22 |  | -0.03 (-0.04,-0.01) |  | 0.47 |  | -0.04 (-0.06,-0.03) |  | 0.86 |
|  |  | >1.48 | 79 | -0.02 (-0.04,-0.01) |  |  |  | -0.03 (-0.05,-0.02) |  |  |  | -0.05 (-0.06,-0.03) |  |  |
|  |  |  |  |  |  |  |  |  |  |  |  |  |  |  |
| **B2-microglobulin, mg/L** | | |  |  |  |  |  |  |  |  |  |  |  |  |
|  | Non-Hispanic | |  |  |  |  |  |  |  |  |  |  |  |  |
|  |  | White | 65 | -0.01 (-0.06, 0.03) |  | 0.25 |  | -0.09 (-0.14,-0.04) |  | 0.35 |  | -0.08 (-0.12,-0.03) |  | 0.52 |
|  |  | Black | 82 | -0.06 (-0.12, 0.00) |  |  |  | -0.05 (-0.12, 0.02) |  |  |  | -0.10 (-0.18,-0.03) |  |  |
|  | Hypertension status | |  |  |  |  |  |  |  |  |  |  |  |  |
|  |  | No | 117 | -0.04 (-0.07,-0.01) |  | 0.91 |  | -0.08 (-0.11,-0.04) |  | 0.72 |  | -0.08 (-0.12,-0.04) |  | 0.61 |
|  |  | Yes | 42 | -0.03 (-0.14, 0.08) |  |  |  | -0.05 (-0.18, 0.07) |  |  |  | -0.11 (-0.23, 0.00) |  |  |
|  | Baseline Triglycerides, mg/dl | |  |  |  |  |  |  |  |  |  |  |  |  |
|  |  | ≤ 83.8 | 80 | -0.06 (-0.12, 0.01) |  | 0.37 |  | -0.06 (-0.13, 0.01) |  | 0.74 |  | -0.12 (-0.19,-0.05) |  | 0.19 |
|  |  | > 83.8 | 79 | -0.02 (-0.06, 0.02) |  |  |  | -0.08 (-0.12,-0.03) |  |  |  | -0.06 (-0.10,-0.02) |  |  |
|  | Body mass index, kg/m^2^ | |  |  |  |  |  |  |  |  |  |  |  |  |
|  |  | 25-29.9 | 69 | -0.07 (-0.14, 0.01) |  | 0.24 |  | -0.09 (-0.18,-0.01) |  | 0.43 |  | -0.13 (-0.21,-0.05) |  | 0.11 |
|  |  | 30+ | 90 | -0.02 (-0.05, 0.02) |  |  |  | -0.06 (-0.09,-0.02) |  |  |  | -0.06 (-0.10,-0.02) |  |  |
|  | Homeostasis Model Assessment (HOMA) | |  |  |  |  |  |  |  |  |  |  |  |  |
|  |  | ≤1.48 | 80 | -0.02 (-0.06, 0.02) |  | 0.38 |  | -0.06 (-0.10,-0.01) |  | 0.55 |  | -0.10 (-0.14,-0.06) |  | 0.72 |
|  |  | >1.48 | 79 | -0.06 (-0.12, 0.01) |  |  |  | -0.08 (-0.16,-0.01) |  |  |  | -0.08 (-0.16,-0.00) |  |  |
|  |  |  |  |  |  |  |  |  |  |  |  |  |  |  |
| **Creatinine, mg/dL** | | |  |  |  |  |  |  |  |  |  |  |  |  |
|  | Non-Hispanic | |  |  |  |  |  |  |  |  |  |  |  |  |
|  |  | White | 65 | -0.01 (-0.02, 0.01) |  | 0.59 |  | 0.01 (-0.01, 0.03) |  | 0.29 |  | 0.00 (-0.01, 0.02) |  | 0.91 |
|  |  | Black | 82 | 0.00 (-0.01, 0.01) |  |  |  | 0.03 (0.01, 0.04) |  |  |  | 0.00 (-0.01, 0.02) |  |  |
|  | Hypertension status | |  |  |  |  |  |  |  |  |  |  |  |  |
|  |  | No | 117 | 0.00 (-0.02, 0.01) |  | 0.57 |  | 0.02 (0.00, 0.03) |  | 0.26 |  | 0.00 (-0.01, 0.02) |  | 0.74 |
|  |  | Yes | 42 | 0.00 (-0.02, 0.03) |  |  |  | 0.03 (0.01, 0.05) |  |  |  | 0.00 (-0.03, 0.02) |  |  |
|  | Baseline Triglycerides, mg/dl | |  |  |  |  |  |  |  |  |  |  |  |  |
|  |  | ≤ 83.8 | 80 | -0.01 (-0.02, 0.01) |  | 0.29 |  | 0.02 (0.01, 0.04) |  | 0.56 |  | 0.00 (-0.02, 0.02) |  | 0.98 |
|  |  | > 83.8 | 79 | 0.01 (-0.01, 0.02) |  |  |  | 0.02 (-0.00, 0.03) |  |  |  | 0.00 (-0.01, 0.02) |  |  |
|  | Body mass index, kg/m^2^ | |  |  |  |  |  |  |  |  |  |  |  |  |
|  |  | 25-29.9 | 69 | 0.00 (-0.01, 0.02) |  | 0.81 |  | 0.03 (0.01, 0.04) |  | 0.52 |  | 0.00 (-0.02, 0.02) |  | 0.59 |
|  |  | 30+ | 90 | 0.00 (-0.02, 0.01) |  |  |  | 0.02 (0.00, 0.03) |  |  |  | 0.00 (-0.01, 0.02) |  |  |
|  | Homeostasis Model Assessment (HOMA) | |  |  |  |  |  |  |  |  |  |  |  |  |
|  |  | ≤1.48 | 80 | 0.00 (-0.02, 0.01) |  | 0.97 |  | 0.03 (0.01, 0.04) |  | 0.39 |  | 0.01 (-0.01, 0.02) |  | 0.49 |
|  |  | >1.48 | 79 | 0.00 (-0.02, 0.02) |  |  |  | 0.01 (-0.00, 0.03) |  |  |  | 0.00 (-0.02, 0.01) |  |  |
|  |  |  |  |  |  |  |  |  |  |  |  |  |  |  |
| **eGFRcys, mL/min/1.73 m^2^** | | |  |  |  |  |  |  |  |  |  |  |  |  |
|  | Non-Hispanic | |  |  |  |  |  |  |  |  |  |  |  |  |
|  |  | White | 65 | 2.02 (0.73, 3.32) |  | 0.98 |  | 3.88 (2.02, 5.74) |  | 0.20 |  | 4.79 (3.03, 6.55) |  | 0.78 |
|  |  | Black | 82 | 2.04 (0.92, 3.17) |  |  |  | 2.39 (1.07, 3.71) |  |  |  | 4.49 (3.27, 5.72) |  |  |
|  | Hypertension status | |  |  |  |  |  |  |  |  |  |  |  |  |
|  |  | No | 117 | 1.98 (1.09, 2.86) |  | 0.78 |  | 3.05 (1.84, 4.25) |  | 0.80 |  | 4.14 (3.04, 5.25) |  | 0.32 |
|  |  | Yes | 42 | 1.71 (-0.05, 3.46) |  |  |  | 2.76 (0.69, 4.83) |  |  |  | 5.34 (3.30, 7.38) |  |  |
|  | Baseline Triglycerides, mg/dl | |  |  |  |  |  |  |  |  |  |  |  |  |
|  |  | ≤ 83.8 | 80 | 2.57 (1.33, 3.81) |  | 0.09 |  | 2.56 (1.03, 4.09) |  | 0.45 |  | 4.57 (3.15, 6.00) |  | 0.82 |
|  |  | > 83.8 | 79 | 1.20 (0.22, 2.18) |  |  |  | 3.36 (1.95, 4.78) |  |  |  | 4.35 (3.01, 5.69) |  |  |
|  | Body mass index, kg/m^2^ | |  |  |  |  |  |  |  |  |  |  |  |  |
|  |  | 25-29.9 | 69 | 1.96 (0.72, 3.20) |  | 0.91 |  | 3.48 (1.82, 5.15) |  | 0.40 |  | 5.06 (3.43, 6.69) |  | 0.30 |
|  |  | 30+ | 90 | 1.86 (0.81, 2.90) |  |  |  | 2.57 (1.24, 3.90) |  |  |  | 4.01 (2.81, 5.20) |  |  |
|  | Homeostasis Model Assessment (HOMA) | |  |  |  |  |  |  |  |  |  |  |  |  |
|  |  | ≤1.48 | 80 | 1.51 (0.53, 2.49) |  | 0.33 |  | 2.66 (1.20, 4.11) |  | 0.55 |  | 4.44 (3.18, 5.69) |  | 0.95 |
|  |  | >1.48 | 79 | 2.32 (1.04, 3.59) |  |  |  | 3.28 (1.77, 4.80) |  |  |  | 4.49 (2.97, 6.00) |  |  |
|  |  |  |  |  |  |  |  |  |  |  |  |  |  |  |
| **eGFRcreat, mL/min/1.73 m^2^** | | |  |  |  |  |  |  |  |  |  |  |  |  |
|  | Non-Hispanic | |  |  |  |  |  |  |  |  |  |  |  |  |
|  |  | White | 65 | 0.91 (-0.70, 2.52) |  | 0.45 |  | -0.55 (-2.26, 1.16) |  | 0.22 |  | 0.25 (-1.19, 1.69) |  | 0.65 |
|  |  | Black | 82 | 0.02 (-1.63, 1.66) |  |  |  | -2.18 (-4.10,-0.25) |  |  |  | -0.33 (-2.38, 1.71) |  |  |
|  | Hypertension status | |  |  |  |  |  |  |  |  |  |  |  |  |
|  |  | No | 117 | 0.50 (-0.75, 1.75) |  | 0.44 |  | -1.08 (-2.57, 0.40) |  | 0.30 |  | 0.16 (-1.09, 1.41) |  | 0.84 |
|  |  | Yes | 42 | -0.48 (-2.68, 1.71) |  |  |  | -2.51 (-4.77,-0.25) |  |  |  | -0.18 (-3.15, 2.79) |  |  |
|  | Baseline Triglycerides, mg/dl | |  |  |  |  |  |  |  |  |  |  |  |  |
|  |  | ≤ 83.8 | 80 | 0.78 (-0.85, 2.42) |  | 0.31 |  | -2.03 (-3.86,-0.20) |  | 0.37 |  | 0.05 (-1.97, 2.08) |  | 0.97 |
|  |  | > 83.8 | 79 | -0.33 (-1.76, 1.10) |  |  |  | -0.91 (-2.58, 0.75) |  |  |  | 0.08 (-1.23, 1.39) |  |  |
|  | Body mass index, kg/m^2^ | |  |  |  |  |  |  |  |  |  |  |  |  |
|  |  | 25-29.9 | 69 | 0.19 (-1.47, 1.85) |  | 0.94 |  | -2.20 (-4.17,-0.23) |  | 0.32 |  | 0.73 (-0.99, 2.45) |  | 0.34 |
|  |  | 30+ | 90 | 0.27 (-1.17, 1.72) |  |  |  | -0.92 (-2.52, 0.68) |  |  |  | -0.44 (-2.12, 1.25) |  |  |
|  | Homeostasis Model Assessment (HOMA) | |  |  |  |  |  |  |  |  |  |  |  |  |
|  |  | ≤1.48 | 80 | -0.06 (-1.52, 1.40) |  | 0.57 |  | -2.09 (-3.78,-0.40) |  | 0.30 |  | -0.60 (-2.41, 1.21) |  | 0.25 |
|  |  | >1.48 | 79 | 0.58 (-1.06, 2.21) |  |  |  | -0.82 (-2.63, 1.00) |  |  |  | 0.77 (-0.82, 2.36) |  |  |
|  |  |  |  |  |  |  |  |  |  |  |  |  |  |  |

Note: eGFRcys represents cystatin C-based estimated glomerular filtration rate; eGFRcreat represents creatinine-based estimated glomerular filtration rate; CG represents the high carbohydrate, high glycemic index diet; cG represents the low carbohydrate, high glycemic index diet; Cg represents the high carbohydrate, low glycemic index diet, and cg represents the low carbohydrate, low glycemic index diet.

| **Supplement Table S2. Between Diet Comparison Restricted to Visit 1 Only** | | | |  |  |  |  |  | |
| --- | --- | --- | --- | --- | --- | --- | --- | --- | --- |
|  | **Mean (95% Confidence Interval)** | | | | | | | | |
|  | **Reducing Glycemic Index**  **(Cg vs. CG)** | |  | **Reducing Carbohydrate (cG vs. CG)** | |  | **Reducing both Glycemic Index and Carbohydrate (cg vs. CG)** | | |
|  | **Difference, 95% CI** | ***P*** |  | **Difference, 95% CI** | ***P*** |  | **Difference, 95% CI** | | ***P*** |
| Cystatin C, mg/L | -0.01 (-0.03, 0.01) | 0.37 |  | -0.03 (-0.05,-0.00) | 0.04 |  | -0.04 (-0.06,-0.02) | | 0.001 |
| B2-microglobulin, mg/L | -0.06 (-0.18, 0.05) | 0.29 |  | -0.11 (-0.24, 0.01) | 0.08 |  | -0.11 (-0.23, 0.01) | | 0.07 |
| Creatinine, mg/dL | 0.01 (-0.02, 0.05) | 0.46 |  | 0.03 (-0.01, 0.07) | 0.16 |  | 0.00 (-0.03, 0.04) | | 0.94 |
| eGFRcys, mL/min/1.73 m^2^ | 1.50 (-1.01, 4.00) | 0.24 |  | 3.35 (0.81, 5.89) | 0.01 |  | 4.25 (1.61, 6.90) | | 0.002 |
| eGFRcreat, mL/min/1.73 m^2^ | -1.51 (-4.58, 1.55) | 0.33 |  | -1.97 (-5.54, 1.60) | 0.28 |  | -0.19 (-3.41, 3.03) | | 0.91 |

Note: eGFRcys represents cystatin C-based estimated glomerular filtration rate; eGFRcreat represents creatinine-based estimated glomerular filtration rate; CG represents the high carbohydrate, high glycemic index diet; cG represents the low carbohydrate, high glycemic index diet; Cg represents the high carbohydrate, low glycemic index diet, and cg represents the low carbohydrate, low glycemic index diet.

| **Supplement Table S3. All Between Diet Comparisons** | |  | |  | |  |  |
| --- | --- | --- | --- | --- | --- | --- | --- |
|  |  |  | |  | |  |  |
|  | **Mean (95% Confidence Interval)** | | | | | | |
|  | **cg vs. cG** | |  | | **Cg vs. CG** | | |
| **Reducing glycemic index** | **β, 95% CI** | ***P*** |  | | **β, 95% CI** | | ***P*** |
| Creatinine, mg/dL | -0.02 (-0.03,-0.01) | **0.004** |  | | 0.00 (-0.01, 0.01) | | 0.92 |
| Cystatin C, mg/L | -0.02 (-0.03,-0.01) | **0.001** |  | | -0.02 (-0.03,-0.01) | | **<0.001** |
| B2-microglobulin, mg/L | -0.02 (-0.06, 0.02) | 0.35 |  | | -0.04 (-0.08,-0.00) | | **0.05** |
| eGFRcys, mL/min/1.73 m^2^ | 1.50 (0.51, 2.48) | **0.003** |  | | 1.90 (1.10, 2.70) | | **<0.001** |
| eGFRcreat, mL/min/1.73 m^2^ | 1.54 (0.25, 2.83) | **0.02** |  | | 0.24 (-0.85, 1.33) | | 0.67 |
| Urine urea nitrogen, mg/d | 117.7 (-560.1, 795.6) | 0.73 |  | | 270.7 (-292.0, 833.5) | | 0.35 |
| Urine creatinine, mg/d | -46.8 (-117.1, 23.4) | 0.19 |  | | -46.2 (-116.5, 24.2) | | 0.20 |
| Urine sodium, mmol/d | -5.9 (-15.1, 3.3) | 0.21 |  | | -7.2 (-16.1, 1.7) | | 0.12 |
| Urine potassium, mmol/d | -2.4 (-7.4, 2.6) | 0.34 |  | | -0.07 (-5.5, 5.4) | | 0.98 |
|  |  |  |  | |  | |  |
|  | **cg vs. Cg** | |  | | **cG vs. CG** | | |
| **Reducing carbohydrate proportion** | **β, 95% CI** | ***P*** |  | | **β, 95% CI** | | ***P*** |
| Creatinine, mg/dL | 0.00 (-0.01, 0.01) | 0.70 |  | | 0.02 (0.01, 0.03) | | **0.001** |
| Cystatin C, mg/L | -0.03 (-0.04,-0.02) | **<0.001** |  | | -0.03 (-0.04,-0.02) | | **<0.001** |
| B2-microglobulin, mg/L | -0.05 (-0.08,-0.02) | **0.001** |  | | -0.07 (-0.11,-0.03) | | **0.001** |
| eGFRcys, mL/min/1.73 m^2^ | 2.57 (1.75, 3.39) | **<0.001** |  | | 2.97 (1.93, 4.01) | | **<0.001** |
| eGFRcreat, mL/min/1.73 m^2^ | -0.17 (-1.27, 0.93) | 0.77 |  | | -1.47 (-2.71,-0.23) | | **0.02** |
| Urine urea nitrogen, mg/d | 3594.9 (2865.8, 4323.9) | **<0.001** |  | | 3747.9 (3002.4, 4493.4) | | **<0.001** |
| Urine creatinine, mg/d | 108.0 (29.7, 186.2) | **0.007** |  | | 108.7 (29.7, 187.7) | | **0.007** |
| Urine sodium, mmol/d | 3.1 (-6.4, 12.7) | 0.52 |  | | 1.9 (-7.8, 11.6) | | 0.71 |
| Urine potassium, mmol/d | -2.8 (-8.1, 2.4) | 0.29 |  | | -0.5 (-5.8, 4.8) | | 0.85 |
|  |  |  |  | |  | |  |
|  | **cG vs. Cg*** | |  | | **cg vs. CG** | | |
| **Changing both glycemic index and carbohydrate proportion** | **β, 95% CI** | ***P*** |  | | **β, 95% CI** | | ***P*** |
| Creatinine, mg/dL | 0.02 (0.01, 0.03) | **0.001** |  | | 0.00 (-0.01, 0.01) | | 0.78 |
| Cystatin C, mg/L | -0.01 (-0.02, 0.00) | **0.02** |  | | -0.04 (-0.05,-0.04) | | **<0.001** |
| B2-microglobulin, mg/L | -0.03 (-0.07, 0.00) | **0.05** |  | | -0.09 (-0.13,-0.05) | | **<0.001** |
| eGFRcys, mL/min/1.73 m^2^ | 1.07 (0.05, 2.08) | **0.04** |  | | 4.47 (3.49, 5.44) | | **<0.001** |
| eGFRcreat, mL/min/1.73 m^2^ | -1.71 (-2.92,-0.50) | **0.006** |  | | 0.07 (-1.14, 1.28) | | 0.91 |
| Urine urea nitrogen, mg/d | 3477.1 (2710.2, 4244.1) | **<0.001** |  | | 3865.6 (3200.7, 4530.5) | | **<0.001** |
| Urine creatinine, mg/d | 154.8 (76.4, 233.3) | **<0.001** |  | | 61.8 (-9.9, 133.6) | | 0.09 |
| Urine sodium, mmol/d | 9.1 (-0.4, 18.5) | 0.06 |  | | -4.1 (-13.2, 5.1) | | 0.38 |
| Urine potassium, mmol/d | -0.4 (-5.5, 4.6) | 0.87 |  | | -2.9 (-8.4, 2.6) | | 0.30 |
| Note: CG represents the high carbohydrate, high glycemic index diet; cG represents the low carbohydrate, high glycemic index diet; Cg represents the high carbohydrate, low glycemic index diet, and cg represents the low carbohydrate, low glycemic index diet. | | | | | | | |
| *The comparison is currently structured to look at low versus high proportion carbohydrate, but could be reversed to look at low versus high glycemic index. | | | | | | | |
